# Observational Study of Repeat Immunoadsorption (RIA) in Post-COVID ME/CFS Patients with Elevated ß2-Adrenergic Receptor Autoantibodies—An Interim Report

**DOI:** 10.3390/jcm12196428

**Published:** 2023-10-09

**Authors:** Elisa Stein, Cornelia Heindrich, Kirsten Wittke, Claudia Kedor, Laura Kim, Helma Freitag, Anne Krüger, Markus Tölle, Carmen Scheibenbogen

**Affiliations:** 1 Institute for Medical Immunology, Charité—Universitätsmedizin Berlin, Corporate Member of Freie Universität Berlin and Humboldt-Universität zu Berlin, Augustenburger Platz 1, 13353 Berlin, Germanykirsten.wittke@charite.de (K.W.); claudia.kedor@charite.de (C.K.); laura.kim@charite.de (L.K.); helma.freitag@charite.de (H.F.); carmen.scheibenbogen@charite.de (C.S.); 2 Department of Nephrology, Charité—Universitätsmedizin Berlin, Corporate Member of Freie Universität Berlin and Humboldt-Universität zu Berlin, Augustenburger Platz 1, 13353 Berlin, Germany; anne.krueger@charite.de (A.K.); markus.toelle@charite.de (M.T.)

**Keywords:** autoantibodies, immunoadsorption, SF-36, physical function, long COVID, GPCR-antibodies, post-COVID syndrome, Myalgic Encephalomyelitis/Chronic Fatigue Syndrome

## Abstract

There is increasing evidence for an autoimmune aetiology in post-infectious Myalgic Encephalomyelitis/Chronic Fatigue Syndrome (ME/CFS). SARS-CoV-2 has now become the main trigger for ME/CFS. We have already conducted two small proof-of-concept studies on IgG depletion by immunoadsorption (IA) in post-infectious ME/CFS, which showed efficacy in most patients. This observational study aims to evaluate the efficacy of IA in patients with post-COVID-19 ME/CFS. The primary objective was to assess the improvement in functional ability. Due to the urgency of finding therapies for post-COVID-Syndrome (PCS), we report here the interim results of the first ten patients, with seven responders defined by an increase of between 10 and 35 points in the Short-Form 36 Physical Function (SF36-PF) at week four after IA. The results of this observational study will provide the basis for patient selection for a randomised controlled trial (RCT), including sham apheresis, and for an RCT combining IA with B-cell depletion therapy. Trial registration number: NCT05629988.

## 1. Introduction

After a mild-to-moderate SARS-CoV-2 infection, approximately 5–10% of patients develop long-lasting symptoms that can be attributed to different conditions and symptom complexes, referred to as Post-COVID-19 Condition or Syndrome (PCS) [1,2]. Common symptoms of PCS include fatigue, cognitive impairment, and pain [3]. About half of PCS patients suffering from moderate-to-severe fatigue and exertion intolerance fulfil the 2003 Canadian Consensus Criteria (CCC) for Myalgic Encephalomyelitis/Chronic Fatigue Syndrome (ME/CFS). Symptoms persisted beyond 20 months post-infection in most patients and encompass the full scope of post-infectious ME/CFS [4]. ME/CFS can be triggered by various infections and is characterised by the core features of fatigue and exercise intolerance with post-exertional malaise (PEM), lasting at least six months after disease onset. PEM is defined as a worsening of symptoms after every-day exertion, often lasting several days or longer [5]. ME/CFS is also characterised by pain, disturbances in sleep, neurocognitive impairment, and the dysregulation of the cardiovascular and immune systems [6]. The mechanisms of PCS are complex and multifactorial, with strong evidence for immune and vascular dysregulation similar to ME/CFS [7,8]. Furthermore, there is evidence of SARS-CoV-2 persistence and clotting abnormalities in PCS [2,9]. Autoimmunity is postulated to play a major role in the pathophysiological mechanisms leading to PCS [10]. Several studies described autoantibodies (AAB) associated with PCS, including AABs to RAS (renin-aldosterone system) proteins, cytokines, antinuclear antibodies (ANA), and other AABs commonly associated with autoimmune diseases [11]. We and others found AABs to G-protein-coupled receptors (GPCR) in PCS to be associated with symptom severity and impaired microcirculation [12,13]. The ß2-adrenergic receptor antibody (ADRB2 AAB) was the best discriminator of PCS, and both fatigue and vasomotor symptoms were strongly associated with the levels of ADRB2 AABs in PCS-ME/CFS patients [13]. These results align with previous findings in post-infectious ME/CFS patients, which described correlations between clinical symptoms, structural central nervous system (CNS) alterations, and levels of AAB against ADRB and other GPCR [14,15,16].

We previously conducted a primary observational study to investigate the effect of immunoadsorption (IA) in patients with infection-triggered ME/CFS with elevated ADRB2 AABs. We observed a rapid improvement of symptoms with both short and long-term responses in seven out of ten patients [17]. Given the clinical and potential pathophysiological overlap of the autoimmune-mediated autonomic nervous system dysfunction between ME/CFS and PCS, the depletion of autoantibodies by IA is thought to be a promising therapeutical approach in PCS [9,18,19].

IA is an apheresis technique used to remove immunoglobulins from a patient’s plasma. Plasma is passed through an absorber that can selectively bind immunoglobulin G (IgG) or all immunoglobulins [20]. The absorber can be regenerated during plasma processing, allowing highly effective removal with few side effects [21]. 

Due to the urgency of finding therapies, we present here an interim analysis of the results from the first 10 patients in our prospective observational IA study of patients with SARS-CoV-2-triggered ME/CFS. Patients with elevated ADRB2 AABs were selected based on the association of the levels with symptom severity [13]. This study is conducted within the Nationale Klinische Studiengruppe (NKSG), a clinical trial and translational research platform for the development of treatment in PCS and ME/CFS, funded by the German Ministry of Education and Research (BMBF) [22]. 

## 2. Materials and Methods

### 2.1. Patients

Patients were diagnosed with ME/CFS based on the 2003 CCC for ME/CFS. Patients were recruited from October 2022 to February 2023 at the Charité Fatigue Centre at the Institute of Medical Immunology, Charité Berlin. Inclusion criteria required having a positive PCR or antigen test for COVID-19 at disease onset and elevated ADRB2 AABs. Other relevant conditions that could cause PCS or fatigue were excluded.

### 2.2. Study Protocol

We conducted an observational study to assess the effect of IA on physical disability, symptom severity, immunoglobulin, and antibody levels. The study was approved by the Ethics Committee of Charité Universitätsmedizin Berlin in accordance with the 1964 Declaration of Helsinki and its subsequent amendments. All patients provided written informed consent. Immunoadsorption was performed using TheraSorb^®^ columns designed for the specific removal of human lambda and kappa chains, including IgG (subclasses IgG1–IgG4), IgA, IgM, and IgE (Miltenyi Biotec B.V. & Co., KG, Bergisch Gladbach, Germany). The IA was performed within the approved use. Five sessions of IA treatments were carried out over a period of ten days, with a maximum of two days in between treatments. Two further IAs will be offered to responding patients who deteriorate again. 

### 2.3. Assessment of Immunoglobulins and Autoantibodies 

IgG, IgA, and IgM levels were measured before IA, before the 5th IA, and four weeks after the first IA. Autoantibodies were measured before IA, after four IAs, and four weeks after the first IA. Antibodies against ADRB2 AABs were determined by CellTrend GmbH, Luckenwalde, Germany, using ELISA technology. Pre- and post-treatment samples were analysed in the same assay run. The upper normal levels of autoantibodies were determined based on validation studies of a healthy control group and defined as being larger than the 90th percentile of a healthy control group (14 U/L).

### 2.4. Assessment of Physical Function and Symptoms 

The primary endpoint was to assess the effect of IA on physical function four weeks after IA by the Short-Form 36 version 2 questionnaire (SF 36), specifically the Physical Function (PF) domain. It has been shown that an increase of at least 10 points in the SF-36 PF scale, which ranges from 0 to 100 and where higher scores indicate better health, indicates a clinically relevant improvement (“a little better”) and an increase of 20 points, a greater clinical improvement (“much better”) [23]. Therefore, we defined an increase of at least 10 points at four weeks after IA as the minimum threshold for considering a patient’s response as positive. In addition, we utilised the Bell score ranging from 0 for severe symptoms to 100 for no symptoms, the Fatigue Severity Scale (FSS) with higher scores indicating more severe fatigue, and weighted CCC symptoms to assess the presence and severity of symptoms [24,25]. The cognitive score and the immune score have been calculated based on the weighted CCC symptoms. The cognitive score has been calculated as the mean of the items for memory disturbance, concentration ability, and mental tiredness and the immune score as the mean of the items for painful lymph nodes, sore throat, and flu-like symptoms, with higher scores indicating more symptoms for both scores [25]. Patient interviews were conducted both before and four weeks after IA, and results of the questionnaires were reviewed for plausibility. 

### 2.5. Data Collection and Management

Study data were collected and managed using REDCap electronic data capture tools hosted at Charité—Universitätsmedizin Berlin [26,27]. 

### 2.6. Statistical Analysis

Statistical data analyses were conducted using GraphPad Prism 9.5.1, © 2023 GraphPad Software (San Diego, CA, USA). Nonparametric statistical methods were used. Continuous variables were presented as median and interquartile range (IQR). Comparisons of different time points of two dependent groups were done using the Wilcoxon matched-paired signed-rank test. A two-tailed *p*-value of <0.05 was considered statistically significant. 

## 3. Results

### 3.1. Patients Characteristics

All patients were diagnosed with post-COVID-19 ME/CFS with a disease duration of nine to 32 months at study inclusion. Age ranged between 33 and 59 years, with six patients being female and four patients being male. Functional disability assessed by the Bell score ranged from 20 to 40, where a score of 100 represents the absence of functional disability, and the SF36-PF score ranged from five to 45, with 100 being the highest possible score. Patient characteristics are presented in Table 1.

### 3.2. Course of IgG, IgA, IgM, and ADRB2 AAB 

**Figure 1:**
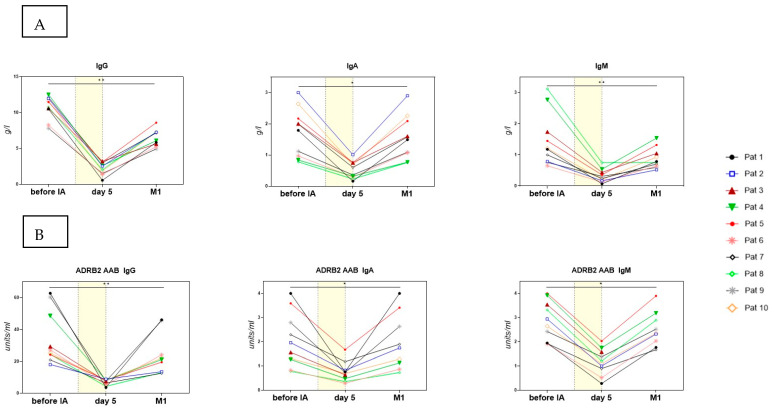
(**A**) Levels of IgG, IgA, and IgM before IA, the morning before the 5th IA, and 4 weeks after IA. (**B**) Levels of ADRB2 AAB IgG, ADRB2 AAB IgA, and ADRB2 AAB IgM before IA, the morning before the 5th IA, and one month (M1) after IA. The period of IA treatment is indicated by a yellow area. ADRB2 AABs were determined by CellTrend GmbH, Luckenwalde, Germany, using ELISA technology. Statistics performed by Wilcoxon matched paired-signed rank test, * *p* < 0.05, ** *p* < 0.01.

In all patients, the total IgG levels were within the normal range (median 11.14 g/L) prior to the first IA and decreased to a median of 2.29 g/L (range 0.57–3.23) after four days of IA. IgA and IgM were within the normal range before IA (median 1.89 g/L (IgA) and 1.19 g/L (IgM)) and decreased to a median of 0.48 g/L and 0.27 g/L, respectively. Four weeks after the first IA, the levels of IgG, IgA, and IgM increased again to a median of 6.64 g/L (IgG), 1.53 g/L (IgA), and 0.77 g/L/(IgM), but they remained significantly lower compared to pretreatment (Figure 1A).

ADRB2 AABs decreased in parallel with the immunoglobulin levels, going from a median of 26.2 U/mL (IgG)/1.8 U/mL (IgA)/2.8 U/mL (IgM) to a median of 7.7 U/mL/0.7 U/mL/1.2 U/mL after four IAs. They then increased again to a median of 21.2 U/mL (IgG)/1.7 U/mL (IgA)/2.4 U/mL (IgM) (Figure 1B). However, we found no correlation in the levels of AABs before or after treatment and the treatment response.

### 3.3. Clinical Course

**Figure 2:**
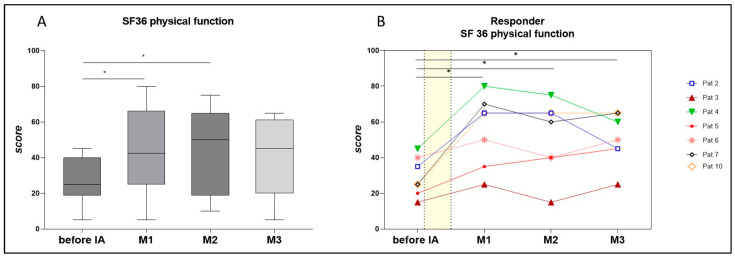
(**A**) Physical function as assessed by the Short Form-36 (SF-36) in all patients (n = 10) before immunoadsorption (IA) and at months (M) 1, 2, and 3 post-IA. A higher score indicates better health. Statistics performed by Wilcoxon matched paired-signed rank test, * *p* < 0.05. (**B**) Physical function as assessed by the SF-36 in responders to IA (n = 7), before IA, and at months 1, 2, and 3 post-IA. Statistics performed by Wilcoxon matched paired-signed rank test, * *p* < 0.05.

The SF36-PF score, defined as the primary outcome parameter, ranged from five to 45 at the baseline (median 25). Seven patients reported an increase in SF36-PF by 10 to 45 points at week 4 after IA (to median 42.5), as shown in Table 1 and Figure 2A for all patients and Figure 2B for individual responding patients. 

As depicted in Figure 2B, four patients reported a rapid and substantial improvement in the SF-36-PF, with an increase of 30 to 45 points at week four after IA (patients 2, 4, 7, and 10). Three patients reported a minor improvement in SF-36 with gains of 10 to 15 points (patients 3, 5, and 6). Notably, patient 5 exhibited a gradual but consistent improvement over the course of three months after IA, starting from 20 before treatment and reaching 40 points at month three. In two patients who initially showed significant improvement after four weeks, we observed a decline in the SF-36-PF at month 3 (patients 2 and 4). 

**Figure 3:**
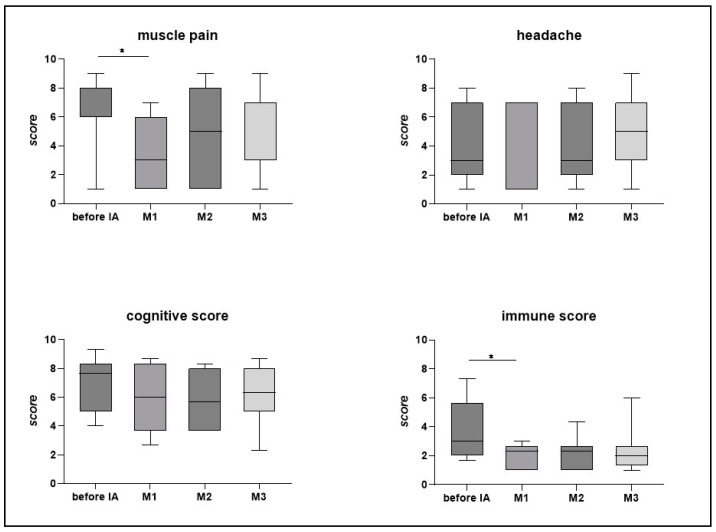
Course of symptoms in responding patients (n = 7): muscle pain, headache, cognitive score, and immune score, as assessed by weighted Canadian Consensus Criteria symptoms. A higher score indicates more severe symptoms. Statistics performed by Wilcoxon matched paired-signed rank test, * *p* < 0.05.

Responders also described improvements in the core symptoms of pain, cognition, and immunological symptoms. Figure 3A shows the progression in the responding patients. Improvements in muscle pain and the immune score were significant after four weeks (*p* < 0.05), while improvements in the cognitive score and headache did not reach statistical significance. However, only four of the seven responders reported headaches before IA. 

**Figure 4:**
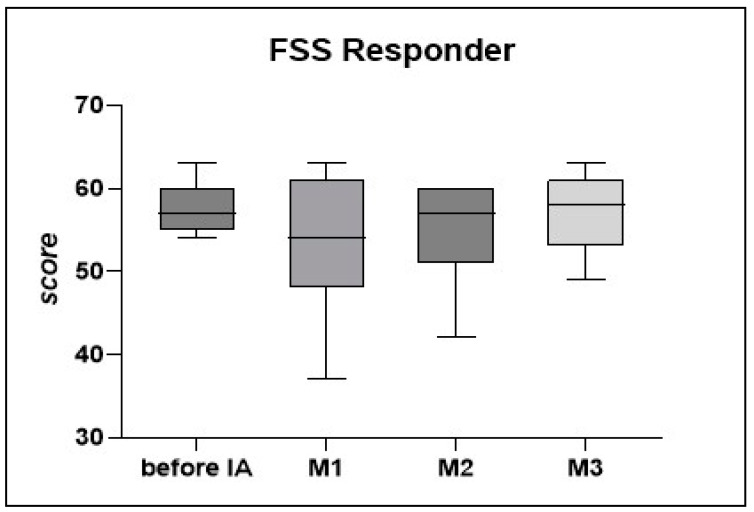
Fatigue in responding patients (n = 7) was assessed by the Fatigue Severity Scale (FSS) before IA and after months 1, 2, and 3. A higher score indicates more severe fatigue. Statistics performed by Wilcoxon matched paired-signed rank test.

Several patients reported an initial worsening of the fatigue alongside PEM, which they attributed to the overall strenuous process of the IA treatment. Fatigue scores assessed by the FSS showed no significant change (Figure 4).

### 3.4. Feasibility of IA

The scheduled therapy of 5 days of IA within 10 days could be carried out in all patients in an outpatient setting and took between 4.5 to 9 h. It could be performed with a peripheral venous catheter in six of the ten patients. Four patients needed a central catheter. The IA treatment, placement of the peripheral or central catheter, and daily travelling were rather stressful and several patients reported the triggering of PEM during the therapy. No further side-effects occurred. To ameliorate the procedure, we paid attention to good hydration and the minimisation of physical and mental stress as far as possible. Lorazepam for up to three days was offered as a supportive therapy.

## 4. Discussion

This interim report on the first 10 patients enrolled in our IA observational study provides first evidence that IA can improve physical function and symptoms in a subset of patients with ME/CFS following SARS-CoV-2 infection. PCS is a complex condition with immune [28] and non-immune mechanisms [29], thus it was important for us to understand if IA can be efficacious in SARS-CoV-2-triggered ME/CFS before including these patients in a randomised sham-controlled trial. The SF-36-PF has been commonly used as a primary endpoint in ME/CFS clinical trials [30] and was found to be suitable to assess the efficacy of IA in this observational study as well. Further we observed a significant improvement in muscle pain and immune symptoms in responding patients at week 4. Fatigue assessed by the FSS was not improved. The discrepancy in SF36-PF and FSS may be related to the fact that fatigue often shows improvement later in the recovery process.

AAB levels significantly decreased after IA in all patients, both responders and non-responders, and increased again after 4 weeks. There was no correlation between the AAB levels and efficacy, and patients with symptom improvement at week four showed a similar recurrence of AAB levels. Thus, mechanisms other than mere AAB depletion most likely account for the improvement in a subset of patients. Among these is the apoptosis of AAB-producing B cells. B cell phenotyping in our previous study provided the first evidence for the effect of IA on memory B cells [17]. GPCR AABs belong to a network of natural AABs that communicate with receptors having both agonistic and antagonistic functions [31]. There is a growing understanding of the role of these AABs in both physiological and pathophysiological processes ranging from autoimmunity to protective roles against the development of immune-mediated diseases [32]. In the case of ADRB2 AABs, we could show that they have an agonistic function in healthy individuals, which is attenuated in ME/CFS [33]. Dysfunctional ADRB2 AABs were shown to be associated with Raynaud’s symptoms in PCS and with brain alterations suggestive of hypoperfusion [13,15]. Therefore, it is tempting to speculate that infection may have triggered dysfunctional GPCR AABs, which disturb receptor function. However, numerous other AABs were shown to be triggered by COVID-19, and we have no direct evidence that the depletion of ADRB2 AABs plays a role in clinical response [11,34]. Besides autoimmunity, several other effects of immunopathogenesis may play a role in PCS, including viral persistence, inflammation, endothelial damage, or micoclotting [35,36,37]. The detailed investigation of these pathomechanisms is the subject of a comprehensive biomarker study accompanying this clinical trial within the NKSG platform [22].

## 5. Conclusions

Taken together, the first data from our study provide evidence that IA has efficacy in a subset of patients and, thus, AABs play an important role in the pathomechanism of SARS-CoV-2-triggered ME/CFS. Limitations of our study include the low number of patients having completed therapy so far and the non-controlled treatment. These results are, however, the basis for recruiting patients with SARS-CoV-2-triggered ME/CFS into an IA RCT with sham apheresis and an RCT combining IA with consecutive B-cell depletion. Further, repeat IA will be performed in this observational trial in responding patients who deteriorate again to learn if this can lead to longer remissions. 

## Figures and Tables

**Table 1:** Patient characteristics and response to treatment.

Patient No.	Gender (m/f)	Age (years)	Time Since COVID-19 (Months)	Bell Score before IA	SF-36 PF before IA	SF-36 PF 4 Weeks Post IA	Responder (Yes/No)
1	m	33	23	30	25	30	no
2	m	59	31	30	35	65	yes
3	f	36	10	20	15	25	yes
4	f	52	23	40	45	80	yes
5	f	59	32	30	20	35	yes
6	m	36	9	30	40	50	yes
7	m	41	25	30	25	70	yes
8	f	37	15	30	40	25	no
9	f	44	14	30	5	5	no
10	f	56	15	40	25	65	yes

IA = immunoadsorption; SF-36-PF = Short Form-36-Physical Function.

## Data Availability

The data presented in this study will be available on request from the corresponding author after completion of the study. Due to the sensitive nature of the data and the ongoing data collection and analysis, the data are not publicly available yet.

## References

[1] Soriano J.B., Murthy S., Marshall J.C., Relan P., Diaz J.V., on behalf of the WHO Clinical Case Definition Working Group on Post-COVID-19 Condition (2022). A clinical case definition of post-COVID-19 condition by a Delphi consensus. Lancet Infect. Dis..

[2] Davis H.E., McCorkell L., Vogel J.M., Topol E.J. (2023). Long COVID: Major findings, mechanisms and recommendations. Nat. Rev. Microbiol..

[3] Premraj L., Kannapadi N.V., Briggs J., Seal S.M., Battaglini D., Fanning J., Suen J., Robba C., Fraser J., Cho S.M. (2022). Mid and long-term neurological and neuropsychiatric manifestations of post-COVID-19 syndrome: A meta-analysis. J. Neurol. Sci..

[4] Legler F., Meyer-Arndt L., Mödl L., Kedor C., Freitag H., Stein E., Hoppmann U., Rust R., Wittke K., Siebert N. (2023). Long-term symptom severity and clinical biomarkers in post-COVID-19/chronic fatigue syndrome: Results from a prospective observational cohort. eClinicalMedicine.

[5] Holtzman C.S., Bhatia S., Cotler J., Jason L.A. (2019). Assessment of Post-Exertional Malaise (PEM) in Patients with Myalgic Encephalomyelitis (ME) and Chronic Fatigue Syndrome (CFS): A Patient-Driven Survey. Diagnostics.

[6] Deumer U.S., Varesi A., Floris V., Savioli G., Mantovani E., Lopez-Carrasco P., Rosati G.M., Prasad S., Ricevuti G. (2021). Myalgic Encephalomyelitis/Chronic Fatigue Syndrome (ME/CFS): An Overview. J. Clin. Med..

[7] Sukocheva O.A., Maksoud R., Beeraka N.M., Madhunapantula S.V., Sinelnikov M., Nikolenko V.N., Neganova M.E., Klochkov S.G., Amjad Kamal M., Staines D.R. (2022). Analysis of post COVID-19 condition and its overlap with myalgic encephalomyelitis/chronic fatigue syndrome. J. Adv. Res..

[8] Joseph P., Singh I., Oliveira R., Capone C.A., Mullen M.P., Cook D.B., Stovall M.C., Squires J., Madsen K., Waxman A.B. (2023). Exercise Pathophysiology in Myalgic Encephalomyelitis/Chronic Fatigue Syndrome and Postacute Sequelae of SARS-CoV-2: More in Common Than Not?. Chest.

[9] Turner S., Khan M.A., Putrino D., Woodcock A., Kell D.B., Pretorius E. (2023). Long COVID: Pathophysiological factors and abnormalities of coagulation. Trends Endocrinol. Metab..

[10] Anaya J.M., Herran M., Beltran S., Rojas M. (2022). Is post-COVID syndrome an autoimmune disease?. Expert. Rev. Clin. Immunol..

[11] Wang E.Y., Mao T., Klein J., Dai Y., Huck J.D., Jaycox J.R., Liu F., Zhou T., Israelow B., Wong P. (2021). Diverse functional autoantibodies in patients with COVID-19. Nature.

[12] Szewczykowski C., Mardin C., Lucio M., Wallukat G., Hoffmanns J., Schroder T., Raith F., Rogge L., Heltmann F., Moritz M. (2022). Long COVID: Association of Functional Autoantibodies against G-Protein-Coupled Receptors with an Impaired Retinal Microcirculation. Int. J. Mol. Sci..

[13] Sotzny F., Filgueiras I.S., Kedor C., Freitag H., Wittke K., Bauer S., Sepulveda N., Mathias da Fonseca D.L., Baiocchi G.C., Marques A.H.C. (2022). Dysregulated autoantibodies targeting vaso- and immunoregulatory receptors in Post COVID Syndrome correlate with symptom severity. Front. Immunol..

[14] Fujii H., Sato W., Kimura Y., Matsuda H., Ota M., Maikusa N., Suzuki F., Amano K., Shin I., Yamamura T. (2020). Altered Structural Brain Networks Related to Adrenergic/Muscarinic Receptor Autoantibodies in Chronic Fatigue Syndrome. J. Neuroimaging.

[15] Kimura Y., Sato W., Maikusa N., Ota M., Shigemoto Y., Chiba E., Arizono E., Maki H., Shin I., Amano K. (2023). Free-water-corrected diffusion and adrenergic/muscarinic antibodies in myalgic encephalomyelitis/chronic fatigue syndrome. J. Neuroimaging.

[16] Gravelsina S., Vilmane A., Svirskis S., Rasa-Dzelzkaleja S., Nora-Krukle Z., Vecvagare K., Krumina A., Leineman I., Shoenfeld Y., Murovska M. (2022). Biomarkers in the diagnostic algorithm of myalgic encephalomyelitis/chronic fatigue syndrome. Front. Immunol..

[17] Scheibenbogen C., Loebel M., Freitag H., Krueger A., Bauer S., Antelmann M., Doehner W., Scherbakov N., Heidecke H., Reinke P. (2018). Immunoadsorption to remove ss2 adrenergic receptor antibodies in Chronic Fatigue Syndrome CFS/ME. PLoS ONE.

[18] Dotan A., David P., Arnheim D., Shoenfeld Y. (2022). The autonomic aspects of the post-COVID19 syndrome. Autoimmun. Rev..

[19] Komaroff A.L., Lipkin W.I. (2023). ME/CFS and Long COVID share similar symptoms and biological abnormalities: Road map to the literature. Front. Med..

[20] Koll R.A. (1998). Ig-Therasorb immunoadsorption for selective removal of human immunoglobulins in diseases associated with pathogenic antibodies of all classes and IgG subclasses, immune complexes, and fragments of immunoglobulins. Ther. Apher..

[21] Fuchs K., Rummler S., Ries W., Helmschrott M., Selbach J., Ernst F., Morath C., Gauly A., Atiye S., Stauss-Grabo M. (2022). Performance, clinical effectiveness, and safety of immunoadsorption in a wide range of indications. Ther. Apher. Dial..

[22] Scheibenbogen C., Bellmann-Strobl J.T., Heindrich C., Wittke K., Stein E., Franke C., Pruss H., Pressler H., Machule M.L., Audebert H. (2023). Fighting Post-COVID and ME/CFS-development of curative therapies. Front. Med..

[23] Brigden A., Parslow R.M., Gaunt D., Collin S.M., Jones A., Crawley E. (2018). Defining the minimally clinically important difference of the SF-36 physical function subscale for paediatric CFS/ME: Triangulation using three different methods. Health Qual. Life Outcomes.

[24] Cotler J., Holtzman C., Dudun C., Jason L.A. (2018). A Brief Questionnaire to Assess Post-Exertional Malaise. Diagnostics.

[25] Freitag H., Szklarski M., Lorenz S., Sotzny F., Bauer S., Philippe A., Kedor C., Grabowski P., Lange T., Riemekasten G. (2021). Autoantibodies to Vasoregulative G-Protein-Coupled Receptors Correlate with Symptom Severity, Autonomic Dysfunction and Disability in Myalgic Encephalomyelitis/Chronic Fatigue Syndrome. J. Clin. Med..

[26] Harris P.A., Taylor R., Thielke R., Payne J., Gonzalez N., Conde J.G. (2009). Research electronic data capture (REDCap)—A metadata-driven methodology and workflow process for providing translational research informatics support. J. Biomed. Inform..

[27] Harris P.A., Taylor R., Minor B.L., Elliott V., Fernandez M., O’Neal L., McLeod L., Delacqua G., Delacqua F., Kirby J. (2019). The REDCap consortium: Building an international community of software platform partners. J. Biomed. Inform..

[28] Merad M., Blish C.A., Sallusto F., Iwasaki A. (2022). The immunology and immunopathology of COVID-19. Science.

[29] Castanares-Zapatero D., Chalon P., Kohn L., Dauvrin M., Detollenaere J., Maertens de Noordhout C., Primus-de Jong C., Cleemput I., Van den Heede K. (2022). Pathophysiology and mechanism of long COVID: A comprehensive review. Ann. Med..

[30] Gaunt D., Brigden A., Metcalfe C., Loades M., Crawley E. (2023). Investigating the factors associated with meaningful improvement on the SF-36-PFS and exploring the appropriateness of this measure for young people with ME/CFS accessing an NHS specialist service: A prospective cohort study. BMJ Open.

[31] Dragun D., Philippe A., Catar R., Hegner B. (2009). Autoimmune mediated G-protein receptor activation in cardiovascular and renal pathologies. Thromb. Haemost..

[32] Riemekasten G., Petersen F., Heidecke H. (2020). What Makes Antibodies Against G Protein-Coupled Receptors so Special? A Novel Concept to Understand Chronic Diseases. Front. Immunol..

[33] Hartwig J., Sotzny F., Bauer S., Heidecke H., Riemekasten G., Dragun D., Meisel C., Dames C., Grabowski P., Scheibenbogen C. (2020). IgG stimulated beta2 adrenergic receptor activation is attenuated in patients with ME/CFS. Brain Behav. Immun. Health.

[34] Cremonesi M., Felicetta A., Cannata F., Serio S., van Beek J.J.P., Bombace S., My I., Zanon V., Catalano C., Papadopoulou V. (2023). Long COVID-19 Cardiac Complications Are Associated With Autoimmunity to Cardiac Self-Antigens Sufficient to Cause Cardiac Dysfunction. Circulation.

[35] Vassiliou A.G., Vrettou C.S., Keskinidou C., Dimopoulou I., Kotanidou A., Orfanos S.E. (2023). Endotheliopathy in Acute COVID-19 and Long COVID. Int. J. Mol. Sci..

[36] Guo L., Appelman B., Mooij-Kalverda K., Houtkooper R.H., van Weeghel M., Vaz F.M., Dijkhuis A., Dekker T., Smids B.S., Duitman J.W. (2023). Prolonged indoleamine 2,3-dioxygenase-2 activity and associated cellular stress in post-acute sequelae of SARS-CoV-2 infection. EBioMedicine.

[37] Peluso M.J., Ryder D., Flavell R., Wang Y., Levi J., LaFranchi B.H., Deveau T.M., Buck A.M., Munter S.E., Asare K.A. (2023). Multimodal Molecular Imaging Reveals Tissue-Based T Cell Activation and Viral RNA Persistence for Up to 2 Years Following COVID-19. medRxiv.

